# An inactivated hand-foot-and-mouth disease vaccine using the enterovirus 71 (C4a) strain isolated from a Korean patient induces a strong immunogenic response in mice

**DOI:** 10.1371/journal.pone.0178259

**Published:** 2017-05-24

**Authors:** Hyun Ju In, Heeji Lim, Jung-Ah Lee, Hye Jin Kim, Jin-Won Kim, Ji-Yeon Hyeon, Sang-Gu Yeo, June-Woo Lee, Jung Sik Yoo, Young Ki Choi, Sang-Won Lee

**Affiliations:** 1 Division of Vaccine Research, Korea National Research Institute of Health, Korea Centers for Disease Control and Prevention, CheongJu, Chungcheongbuk-do, Republic of Korea; 2 College of Medicine and Medical Research Institute, Chungbuk National University, CheongJu, Chungcheongbuk-do, Republic of Korea; University of Georgia, UNITED STATES

## Abstract

Enterovirus 71 (EV71) is a major causative agent of hand-foot-and-mouth disease (HFMD) frequently occurring in children. HFMD induced by EV71 can cause serious health problems and has been reported worldwide, particularly in the Asia-Pacific region. In this study, we assessed the immunogenicity of a formalin-inactivated HFMD vaccine using an EV71 strain (FI-EV71 C4a) isolated from a Korean patient. The vaccine candidate was evaluated in mice to determine the vaccination doses and vaccine schedules. BALB/c mice were intramuscularly administered 5, 10, or 20 μg FI-EV71 vaccine, followed by a booster 2 weeks later. EV71-specific antibodies and neutralizing antibodies were induced and maintained until the end of the experimental period in all vaccinated groups. To determine the effectiveness of adjuvant for the EV71 vaccine, three adjuvants, i.e., aluminium hydroxide gel, monophosphoryl lipid A, and polyinosinic-polycytidylic acid, were administered separately with the FI-EV71 vaccine to mice via the intramuscular route. Mice administered the FI-EV71 vaccine formulated with all three adjuvants induced a significantly increased antibody response compared with that of the single adjuvant groups. The vaccinated group with triple adjuvants exhibited more rapid induction of EV71-specific and neutralizing antibodies than the other groups. These results suggested that the role of adjuvant in inactivated vaccine was important for eliciting effective immune responses against EV71. In conclusion, our results showed that FI-EV71 was a potential candidate vaccine for prevention of EV71 infection.

## Introduction

Hand-foot-and-mouth disease (HFMD) is an emerging human infectious disease that frequently occurs in young children under 3 years of age [1]. Most cases of HFMD do not result in serious complications; however, infection with enterovirus 71 (EV71) can cause a high rate of neurologic complications, including meningoencephalitis and pulmonary edema [2, 3]. EV71 is a group of viruses that belongs to the *Picorvaviridae*, it is a non-enveloped, positive, single-stranded RNA virus and four structural capsid proteins, including VP1, VP2 and VP3 on the external surface of the virion and VP4 within the interior of the viral particle [4]. EV71 is currently classified into 3 genotype, A, B and C by VP1. Genotype B and C are further divided into B1-B5 and C1-C5 genotypes. Since 1969, when EV71 was first described, outbreaks of HFMD caused by EV71 have occurred worldwide, particularly in the Asia-Pacific regions [5, 6]. Large outbreaks in China, Japan, Malaysia, Singapore, Taiwan, and Vietnam have been reported in cyclical epidemics every 2 or 3 years. EV71 has also continued to circulate in Africa, Europe, and the USA and causes sporadic cases or small outbreaks [6].

Several EV71 vaccine candidates, including inactivated virus vaccines, attenuated live virus vaccines, subunit vaccines, DNA vaccines, and virus-like particle vaccines, have been developed [7]. Inactivated virus vaccines are the leading strategy for developing vaccines against enteroviruses, as exemplified by the successful commercialization of inactivated poliovirus vaccines [8, 9]. Inactivated virus vaccines have the advantage of conservation of linear and conformational epitopes required for the humoral and cellular immune response. Several inactivated vaccine candidates have been developed and achieved the phase Ⅲ clinical trials [10–13]. Previous studies have confirmed the efficacy of inactivated virus vaccines against EV71, and clinical trials are already being performed in China, Taiwan, and Singapore [13–17].

Inactivated virus vaccines consist of virus particles that are cultured using animal cells and killed by chemical or physical methods. In order to induce antigenicity, it is necessary to use adjuvants and multiple inoculations with inactivated virus vaccines. Adjuvants are used to enhance the immune response against vaccines, resulting in higher amounts of antibodies and long lasting protection. Several different classes of adjuvants have been developed that can promote the immune response. Aluminium hydroxide gel (alum), MF59, and virosomes are licensed for use in human vaccines [18]. Additionally, polycytidylic acid (poly I:C), CpG, imidazoquinolines, and flagellin have been tested in humans but are not yet licensed for use [18]. Alum potentiates the immune response, thereby ensuring the potency and efficacy of typically sparingly available antigen. Alum is most commonly used in licensed vaccines due to enhancement of T helper (Th) 1-type immunity [19]. Monophosphoryl lipid A (MPLA) is a derivative of lipopolysaccharide (LPS), a component of gram-negative bacterial cell membranes, and stimulates toll-like receptor (TLR) 4 signaling [20, 21]. Poly I:C is a synthetic analog of double-stranded RNA, which is used to mimic viral infections [22]. Moreover, poly I:C is associated with TLR3, which is expressed in the membranes of B-cells, macrophages, and dendritic cells.

In this study, formalin-inactivated EV71 vaccine (FI-EV71) derived from a patient who showed clinical signs of HFMD and belonged to the C4 subgenogroup was developed, and the efficacy of FI-EV71 with various adjuvants was evaluated in mice. Our results provide a foundation for the development of inactivated EV71 whole-virus vaccines.

## Materials and methods

### Ethic statements

This study was carried out in strict accordance with the recommendations in the Guide for the are and Use of Laboratory Animals of the National Institutes of Health The animal protocol used in this study has been reviewed by the Institutional Animal Care and Use Committee (IACUC) on their ethical procedures and scientific care and approved (Approval Number KCDC-024-16-2A).

### Cells and viruses

Vero cells were obtained from American Type Culture Collection (ATCC, Manassas, VA, USA) and cultured in Dulbecco’s modified Eagle medium (DMEM) supplemented with 10% fetal bovine serum (FBS) and a penicillin-streptomycin solution. The Vero cells were used to prepare EV71 virus as antigen and to measure the viral titer. The diverse subgenogroups of EV71 (A, B3, C2, C3, and C5) were used for neutralizing assays. Virus titers were determined by microtitration using Vero cells and expressed as the 50% tissue culture infectious dose (TCID_50_), according to the Reed-Muench method.

### Production of inactivated EV71 vaccine

EV71 C4a-89J, the vaccine strain used in this study, was isolated from a patient with HFMD in Korea in 2012. The EV71 vaccine strain belonged to subgenogroup C4 based on VP1 sequence analysis. The virus was initially isolated in human rhabdomyosarcoma (RD) cells and propagated in Vero cells. Before use as a vaccine, the virus was inactivated by adding 0.2% formalin and incubated at 37°C for 5 days. The amount of virion protein was quantified by the Bradford method. FI-EV71 was mixed with 500 μg for alum, 2 μg for MPLA, and 10 μg for poly I:C adjuvant.

### Mouse immunization

To determine the dose of FI-EV71, female BALB/c mice (6 weeks of age) were randomly divided into four groups (n = 6 per group). Each group of mice was intramuscularly administered FI-EV71 (5, 10, or 20 μg) or phosphate-buffered saline (PBS) as a control. FI-EV71 was mixed with alum as an adjuvant at a 1:1 ratio (0.5 mg/mouse). Two weeks after the primary vaccination, all mice were given a booster using the same vaccines. Serum samples were collected at 2, 4, 6, and 8 weeks after primary vaccination (WPV) for assessment of the humoral immune response. Two mice from each group were sacrificed at 3 WPV, and the splenocytes were isolated for analysis of the cell-mediated immune response.

To evaluate the efficacy of FI-EV71 by diverse adjuvants, female BALB/c mice (5 weeks of age, n = 5 per group) were used. The mice were randomly divided into five groups: MOCK, 10 μg EV71 antigen with alum adjuvant (500 μg), 10 μg EV71 antigen with MPLA adjuvant (2 μg), 10 μg EV71 antigen with poly I:C adjuvant (10 μg), and 10 μg EV71 antigen with a combination of all three adjuvants. Mice were intramuscularly administered with FI-EV71 with each adjuvant and triple adjuvants or PBS. Two weeks after the primary vaccination, the mice were administered a booster using the same vaccines. Serum samples were collected at 4, 8, 12, and 16 WPV for serology tests and stored at −80°C. Two mice from each group were sacrificed at 3 WPV, and splenocytes of the mice were isolated for analysis of the cell-mediated immune response.

### Measurement of the EV71-specific IgG antibody response

The EV71-specific IgG antibody response in mouse serum was determined by enzyme-linked immunosorbent assay (ELISA), as described previously [17, 23], with slight modifications. The EV71 vaccine strain (100 ng/100 μL) diluted in coating buffer (0.1 M sodium carbonate) was used as a coating antigen in 96-well microplates (Corning Costar) at 4°C overnight. After blocking with 5% skim milk powder in PBS at 37°C for 2 h, 100 μL serum diluted 1:100 in dilution buffer (2.5% skim milk in PBS) was added to the microplates. After 2 h incubation at 25°C, 100 μL horseradish peroxidase (HRP)-conjugated goat anti-mouse IgG (H+L) antibody (Novex) diluted 1:2000 in dilution buffer was added, and samples were then incubated at 37°C for 1 h. After washing, 50 μL of 3,3’,5,5’-tetra-methylbenzidine (TMB) solution (Rockland) was added for development, followed by the addition of 100 μL of 2 M H_2_O_4_ to stop the reaction. Absorbance was measured at 450 nm using a microplate reader.

The profile of EV71-specific IgG isotypes was determined by ELISA using virus-coated plates, as described above. All protocols were similar, with the exception of the secondary antibodies, which were HRP-conjugated goat anti-mouse IgG1, IgG2a, IgG2b, and IgG3 (Southern Biotech, Birmingham, AL).

### Neutralization antibody assays

Vero cells (7.5 × 10^3^ cells) were seeded in 96-well microplates 1 day before the test. Serum samples from mice were two-fold serially diluted and mixed with equal volumes of EV71 vaccine strain (100 TCID_50_) at 37°C for 2 h. Virus-serum mixtures were added to the Vero cells and incubated at 37°C for 7 days. The neutralization antibody titers were determined as the highest serum dilutions that exhibited a 50% reduction in the cytopathic effect (CPE).

### Cytokine and chemokine analysis

Splenocytes from vaccinated mice were isolated and treated with red blood cell (RBC) lysis buffer (0.84% NH_4_Cl solution) to remove RBCs. After several washes with PBS, 2 × 10^6^ splenocytes were cultured in 24-well plates with RPMI 1640 medium containing 10% FBS, 1% penicillin, and 1% streptomycin. The splenocytes were stimulated with 0.1 multiplicity of infection (MOI) of EV71 C4a-89J and incubated for 48 h. The expression of cytokines and chemokines was examined using a Bio-plex Pro Mouse 23-plex kit (Bio-Rad, Hercules, CA, USA) according to the manufacturer’s instructions and were analyzed using Bio-Plex Manager software (Bio-Rad).

### Statistics

All statistical analyses were performed with GraphPad Prism version 5. Data were analyzed using one-way analysis of variance (ANOVA), followed by Tukey’s post-hoc test.

## Results

### Determination of the FI-EV71 dosage

**Fig 1:**
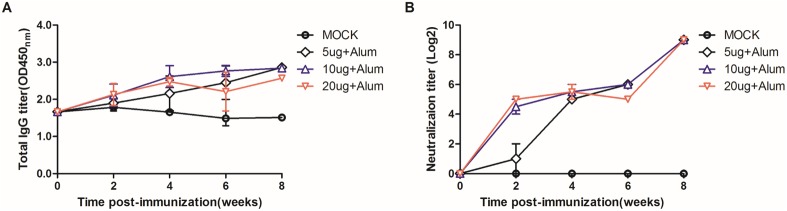
EV71-specific humoral immune responses induced by a formalin-inactivated EV71 vaccine in mice. Mice were administered inactivated EV71 vaccine mixed with 500 μg for alum, 2 μg for MPLA, and 10 μg for poly I:C adjuvant or PBS as a control. (A) Titers of total IgG antibodies against EV71 viral particles were determined by ELISAs. (B) The levels of neutralization antibodies against EV71 were measured by endpoint dilution of serum in neutralization assays.

To determine whether FI-EV71 elicited a humoral immune response against EV71, the immunogenicity was assessed by ELISAs and neutralization antibody assays. EV71-specific IgG antibodies were induced in all group at 2 WPV (Fig 1A). The anti-EV71 IgG titers were gradually increased following booster immunization and remained constant until the end of the experiment.

Regardless of the antigen dosage, all groups of mice showed significantly increased neutralization antibody titers compared with those of control mice (Fig 1B). The neutralization antibody titers in mice vaccinated with 10 and 20 μg EV71 antigen were induced as early as 2 WPV when the mice were vaccinated once. After booster vaccination, the neutralization antibody titers of all groups were increased and maintained at a constant level until the end of the experiment.

**Fig 2:**
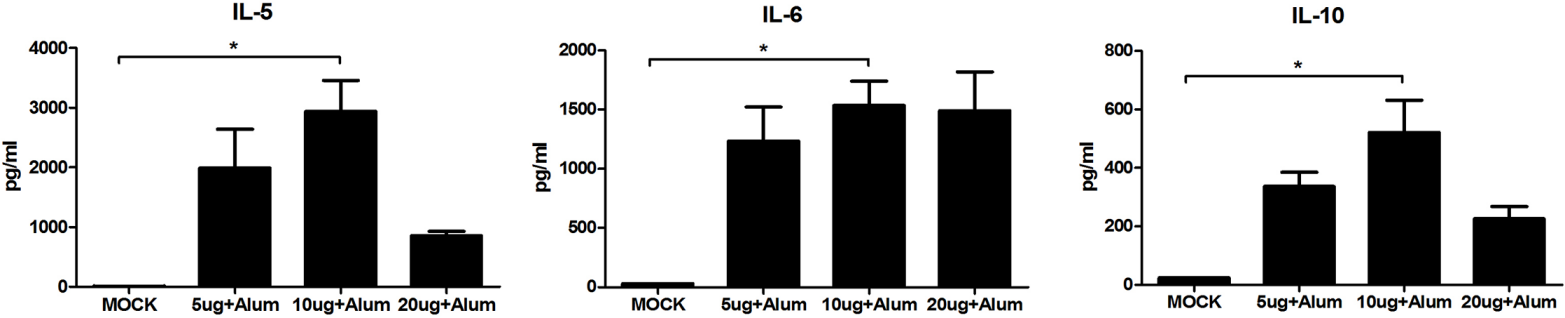
EV71-specific cell mediated immune responses induced by a formalin-inactivated EV71 vaccine in mice. Mice were administered inactivated EV71 vaccine mixed with 500 μg for alum, 2 μg for MPLA, and 10 μg for poly I:C adjuvant or PBS as a control. Splenocytes from vaccinated mice were isolated and stimulated with the EV71 vaccine. The supernatants of splenocytes were harvested at 48 h after stimulation and analyzed for cytokines using a multiplex system. Graphs show means + SEMs. *p < 0.01, using one-way ANOVA with Tukey’s post-hoc test.

Splenocytes from vaccinated mice were used to measure induced cytokines by employing a multiplex system. Interleukin (IL)-5, IL-6, and IL-10 were significantly increased in vaccinated mice, particularly in the 10 μg antigen dose group, compared with that in the MOCK group (Fig 2). These results suggested that 10 μg FI-EV71 antigen was the most effective dose for rapid induction of humoral and cell-mediated immune responses against EV71.

### Immunogenicity of FI-EV71 combined with different adjuvants

**Fig 3:**
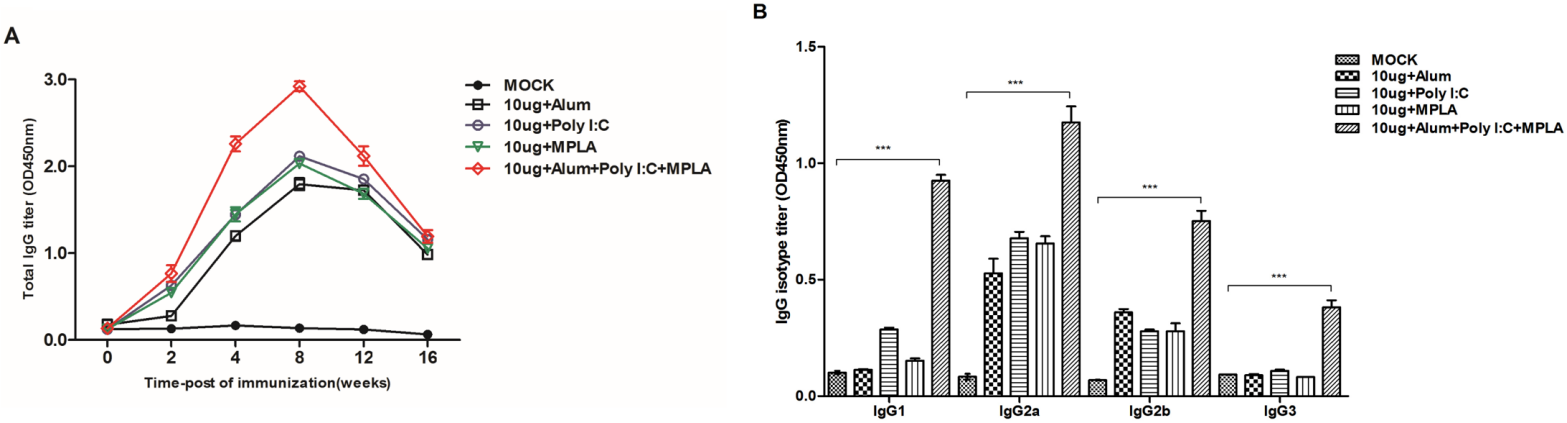
EV71-specific immune responses in serum samples from mice vaccinated with the formalin-inactivated EV71 vaccine combined with diverse adjuvants. Mice were administered the inactivated EV71 vaccine mixed with 500 μg for alum, 2 μg for MPLA, and 10 μg for poly I:C adjuvant. (A) The lgG antibody titer against EV71 was measured by ELISA at 4, 8, 12 and 16 weeks. (B) IgG isotype analysis of EV71-specific IgGs in sera of mice at 8 weeks postvaccination. The IgG isotype graph shows means + SEMs. ***p < 0.0001 using one-way ANOVA with Tukey’s post-hoc test.

Vaccination with FI-EV71 combined with different adjuvants elicited antibody responses in mice. The EV71-specific antibody titer of the group vaccinated with FI-EV71 combined with all three adjuvants was much higher than those of the groups vaccinated with single adjuvants (Fig 3A). In contrast, titers in the MOCK group as a control remained at baseline after vaccination. Analysis of EV71-specific IgG isotypes indicated that the IgG2a and IgG2b responses were increased in all vaccination groups (Fig 3B). In particular, the triple adjuvant group showed a higher IgG isotype response (*p* < 0.0001) than all other groups. These results indicated that FI-EV71 combined with adjuvants triggered a mixed Th1/Th2 response.

**Fig 4:**
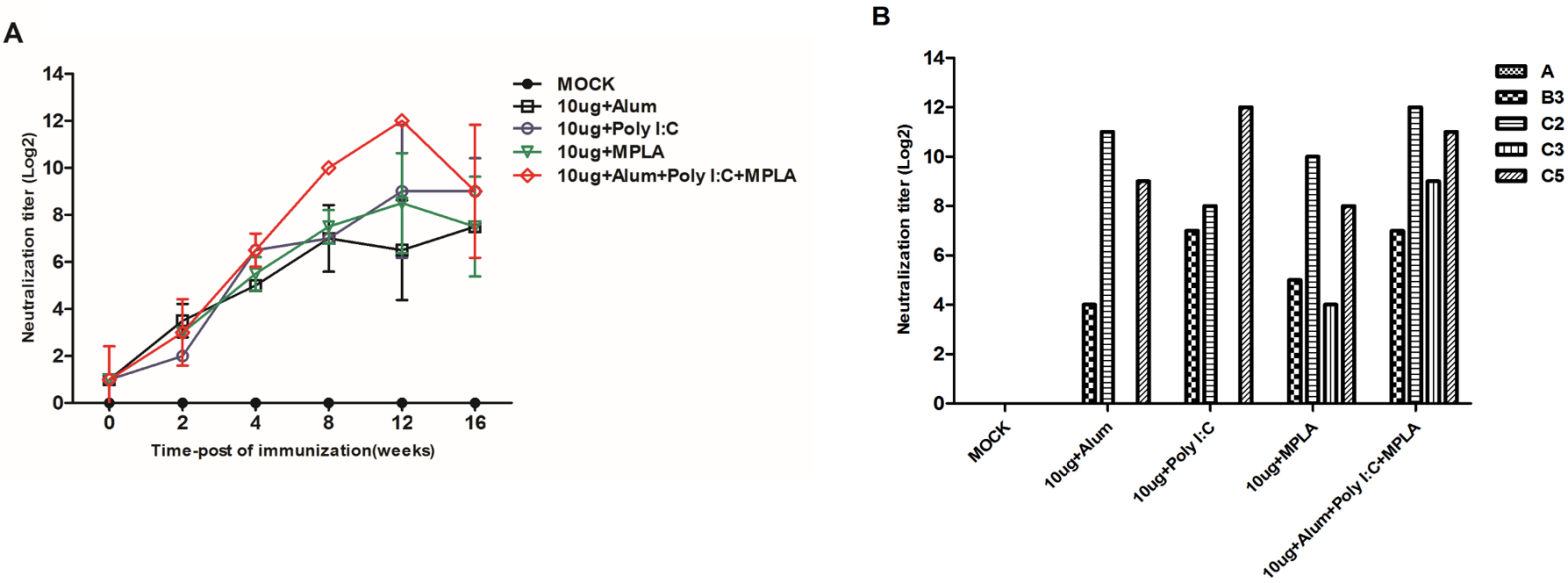
EV71-specific immune responses in serum samples from mice vaccinated with the formalin-inactivated EV71 vaccine combined with diverse adjuvants. Mice were administered the inactivated EV71 vaccine mixed with 500 μg for alum, 2 μg for MPLA, and 10 μg for poly I:C adjuvant and measurement of neutralization antibody titer(A) and cross-reactivity of neutralization antibodies (B) by heterologous EV71 strains. Crossreactivity was determined using sera from mice at 16 weeks postvaccination. ***p < 0.0001 using one-way ANOVA with Tukey’s post-hoc test.

The EV71-specific neutralization antibody titer was found to increase quickly after vaccination in all vaccinated groups, whereas the MOCK group did not show any neutralization antibody responses (Fig 4A). To investigate whether FI-EV71 could induce crossreactive neutralization antibody responses by heterologous EV71 strains, five different EV71 strains (A, B3, C2, C3, and C5) were evaluated. Crossreactive neutralization antibody responses against subgenogroups B3, C2, and C5 were elicited in all adjuvant groups (Fig 4B), and subgenogroup C3 was found in FI-EV71 with MPLA and triple adjuvant groups. In contrast, there were no crossreactive neutralizing antibody responses against subgenogroup A.

**Fig 5:**
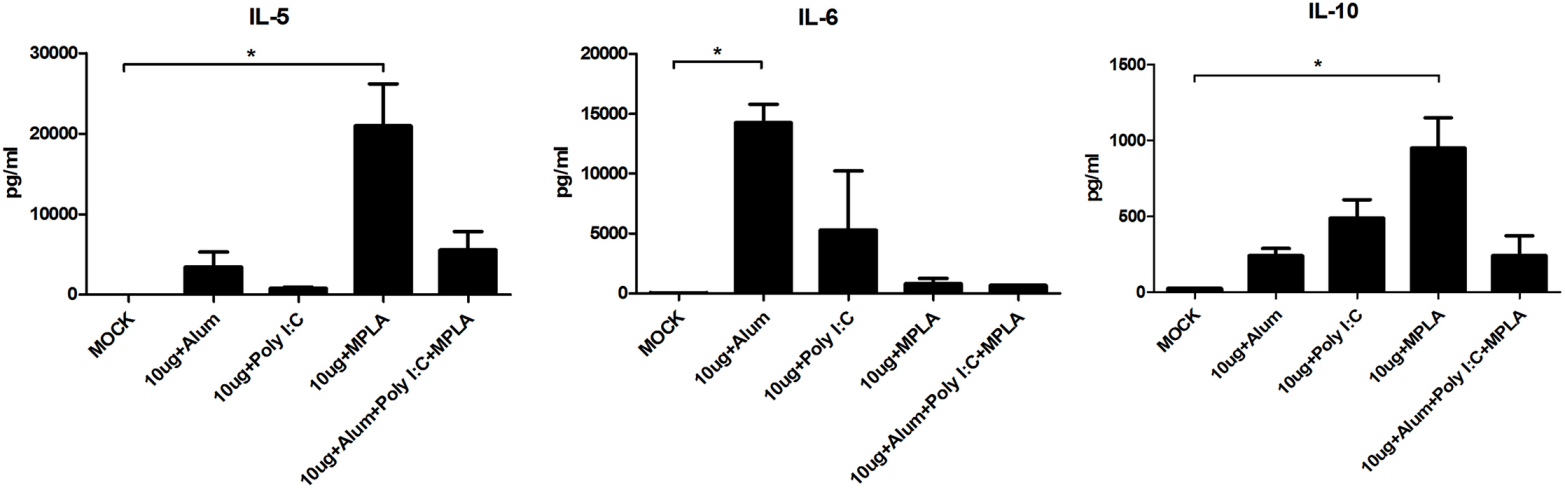
EV71-specific cell mediated immune responses induced by the formalin-inactivated EV71 vaccine with diverse adjuvants. Splenocytes from vaccinated mice were isolated and stimulated with the EV71 vaccine strain. Supernatants of splenocytes were harvested at 48 h after stimulation and analyzed for cytokine levels using a multiplex system. Graphs show means + SEMs. *p < 0.01, using one-way ANOVA with Tukey’s post-hoc test.

To investigate cell-mediated immune responses, splenocytes from mice vaccinated with FI-EV71 combined with different adjuvants were isolated and used to measure induced cytokines. IL-5, IL-6, and IL-10 were significantly increased in vaccinated mice compared with those in unvaccinated mice as a negative control (Fig 5). In particular, mice vaccinated with alum or MPLA induced higher levels of IL-5, IL-6, and IL-10. These results indicated that the Th2 immune response was elicited by the adjuvants alum and MPLA.

## Discussion

EV71 consists of diverse genogroups (A, B1–5, and C1–5). Among them, the predominant subgenogroup in Korea and China is C4 [24, 25]. In this study, the EV71 isolate belonging to the C4 subgenogroup was used to produce a formalin-inactivated vaccine, FI-EV71, and the efficacy according to antigen dosage and diverse adjuvants was evaluated. FI-EV71 induced specific immune responses in terms of high levels of neutralization antibody titers and IgG antibody titers in all groups of vaccinated mice administered 5, 10, or 20 μg antigen. In particular, mice administered 10 and 20 μg FI-EV71 showed rapid induction of neutralization antibody titers by single vaccination. The cell-mediated immune response against FI-EV71 was also induced, as demonstrated by high levels of cytokines in mice vaccinated with an antigen dose of 10 μg. Our current findings suggested that 10 μg FI-EV71 antigen resulted in optimal vaccine efficacy.

Inactivated vaccines can have low immunogenicity following vaccination, particularly when the viral antigen is used alone. However, combination of the inactivated vaccine with a suitable adjuvant enhances the immunogenicity of the vaccine [26]. In this study, the effective adjuvant properties of FI-EV71 were evaluated in the vaccination of mice against EV71. The group administered FI-EV71 formulated with poly I:C adjuvant induced higher antibody titers than other groups vaccinated with a single adjuvant (alum or MPLA). Thus, the addition of poly I:C was critical for boosting neutralization antibodies against EV71. Moreover, administration of FI-EV71 with all three adjuvants strongly stimulated the production of systemic antibodies compared with that using single adjuvants.

Recent epidemics of HFMD have been reported to be caused by diverse subgenogroups of EV71 in Malaysia (B3 in 1997, B4 in 2000), Taiwan (C2 in 1998, B4 in 2000), Singapore (B4 in 2000), Vietnam (C5 in 2005), China (C4 in 2008), and Korea (C3 in 2000, C4 in 2009) [27]. Thus, it is important for an EV71 candidate vaccine to induce crossreactive neutralization antibodies against heterologous EV71 strains. In the present study, FI-EV71 with adjuvant exhibited significant crossreactivity to B3, C2, C3, and C5, and the crossreactive neutralization antibody titers of mice vaccinated with FI-EV71 combined with all three adjuvants were higher than those of any other group. Taken together, these results indicated that FI-EV71 combined with adjuvants had broad protection potential against diverse enterovirus strains.

Several adjuvants for vaccination have been licensed in humans; in particular, the adjuvant alum has been widely used for many years [28]. In previous studies, diverse adjuvant mixtures were used in human vaccines, and the efficacy of these adjuvants was approved [29]. The AS01 adjuvant (GlaxoSmithKline), a combination of the immunostimulant MPL (the clinical grade form of MPLA) and the saponin QS-21, is used in licensed or candidate human vaccines [30]. The AS01 adjuvant directly affects the innate immune response to orchestrate the quality and intensity of the adaptive immune response to vaccine antigens [31]. AS03 (squalene, Tween 80, and α-tocopherol) and AS04 (alum and MPL) adjuvants are the only adjuvant combinations licensed for use in human vaccines [18]. AS03 is currently licensed for pandemic influenza vaccines and is used worldwide [32]. AS03 has been successfully used to enhance the efficacy, immunogenicity, and crossprotection of pandemic influenza vaccines. Recently, the adjuvant formulation AS25 (AS03, MPL) was shown to induce greater immunogenicity for the influenza vaccine than AS03 [33]. Another combination adjuvant, AS04, is approved for use with hepatitis B virus (HBV) and human papilloma virus (HPV) vaccines. When combined with AS04, the HPV vaccine induces higher antibody titers and long-term immune responses than that when the vaccine includes only alum adjuvant, as demonstrated in clinical trials [34]. The crossreactivity to heterologous HPV strains was also enhanced by AS04. Although the efficacy of combination adjuvants is higher than that of single adjuvants, the selection of suitable adjuvants for antigens is important for enhancing the antigen-specific immune response. Enhanced cytokine production is thought to contribute to EV71 pathogenesis in humans and mice [35–37]. Secretion of cytokines and chemokines after EV71 infection from patients with HFMD is essential for developing effective protection against the disease [36–38]. Th cells, particularly Th1 and Th2 cells, play a major role in humoral and cellular immune responses. Th1 cells induce the cytokines interferon (IFN)-γ and tumor necrosis factor (TNF)-α, which are capable of promoting T-cell cytotoxicity. Th2 cells have the capacity to produce IL-4, IL-5, IL-6, IL-10, and IL-13, which enhance the B-cell response. Activation of Th1 cells enhances the production of IgG2a, whereas IgG1 is produced by Th2 cells [38]. In general, inactivated virus vaccines induce the Th2 immune response via B-cell stimulation. In this study, FI-EV71 with adjuvant induced high levels of the IgG2a response. Moreover, cytokine analysis showed that several cytokines, including IL-5, IL-6, and IL-10, which are associated with Th2 cells, were elevated in the vaccinated group. Overall, FI-EV71 with adjuvants triggered a mixed Th1/Th2 response.

In summary, the formalin-inactivated EV71 vaccine FI-EV71 significantly enhanced immunogenicity against EV71. Currently, clinical trials of inactivated EV71 vaccine are being performed in China, Taiwan, and Singapore [13]. The safety of the inactivated EV71 vaccine has already been confirmed in humans [13, 39]. In this study, we showed that an EV71 vaccine combined with multiple adjuvants elicited strong immune responses in mice. However, the cell-mediated immune response of vaccinated mice using FI-EV71 with alum or MPLA as adjuvants was higher than that induced using all three adjuvants. Thus, furthers studies are needed to determine the optimal adjuvant(s) for FI-EV71 and to analyze efficacy in nonhuman primates in order to assess the potential of FI-EV71 as a vaccine candidate for humans. These results will provide valuable information for the development of an inactivated EV71 vaccine.

## Supporting information

S1 File The neutralizing antibody responses by a formalin-inactivated EV71 vaccine in mice. (XLSX)
